# Supplementary figures and images for: Augmin deficiency in neural stem cells causes p53-dependent apoptosis and aborts brain development
Source: eLife. 2021 Aug 24;10:e67989. doi: 10.7554/eLife.67989 (PMC8456695; doi:10.7554/eLife.67989)

Figure 1-figure supplement 1-Source Data 1

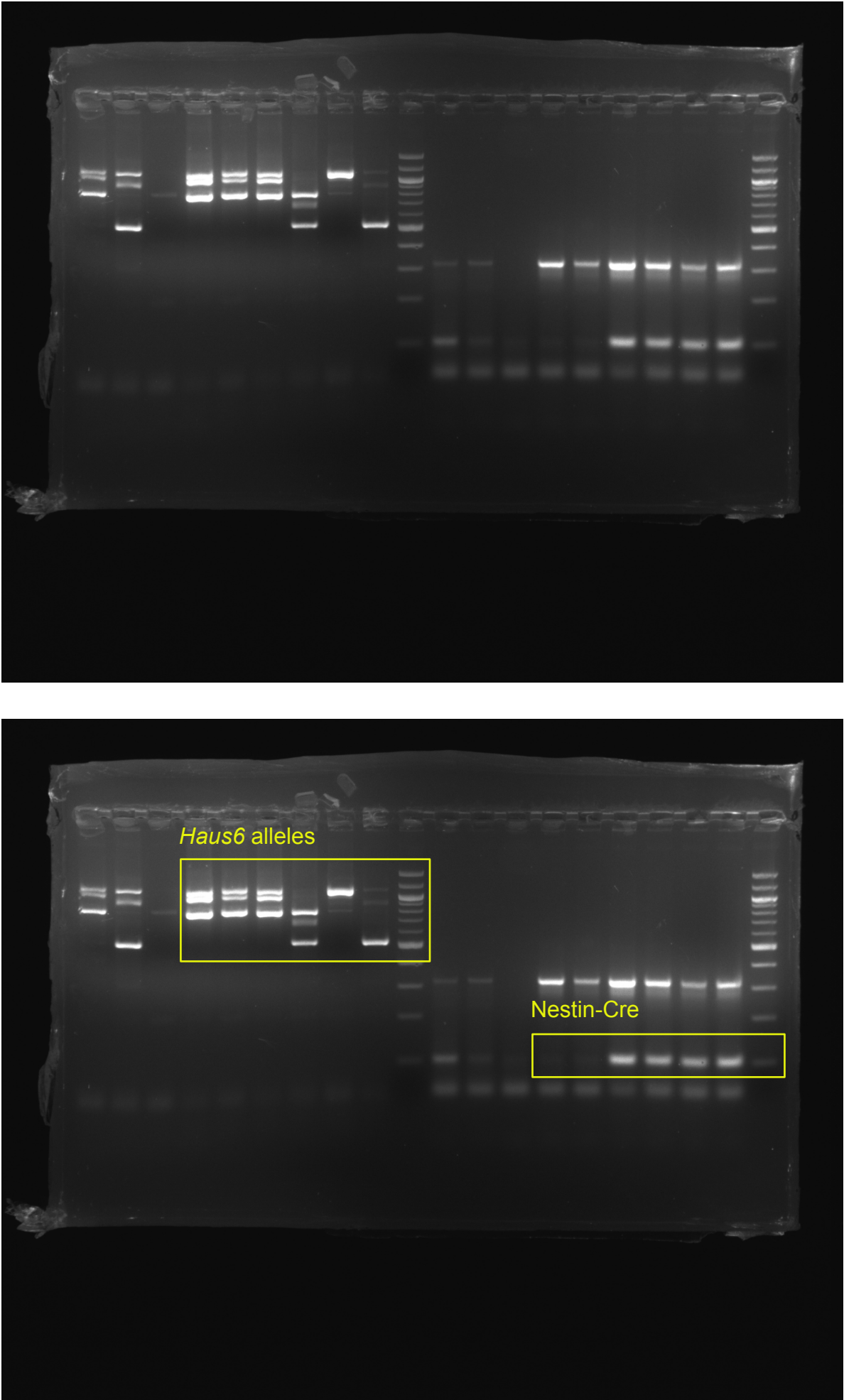

Supplement: Figure 1—figure supplement 1—source data 1. [file elife-67989-fig1-figsupp1-data1.pdf]
